# Pseudo-backcrossing design for rapidly pyramiding multiple traits into a preferential rice variety

**DOI:** 10.1186/s12284-014-0035-0

**Published:** 2015-02-05

**Authors:** Siriphat Ruengphayak, Ekawat Chaichumpoo, Supaporn Phromphan, Wintai Kamolsukyunyong, Wissarut Sukhaket, Ekapol Phuvanartnarubal, Siripar Korinsak, Siriporn Korinsak, Apichart Vanavichit

**Affiliations:** Rice Science Center, Kasetsart University, Kamphaeng Saen, Nakhon Pathom 73140 Thailand; Interdisciplinary Graduate Program in Genetic Engineering, Kasetsart University, Chatuchak, Bangkok 10900 Thailand; Rice Gene Discovery, National Center for Genetic Engineering and Biotechnology (BIOTEC) National Science and Technology Development Agency (NSTDA), Kasetsart University, Kamphaengsaen, Nakhon Pathom 73140 Thailand; Agronomy Department, Faculty of Agriculture at Kamphaeng Saen, Kasetsart University, Kamphaeng Saen, Nakhon Pathom 73140 Thailand

**Keywords:** Gene pyramiding, Multiple backcrossing, Submergence tolerance, Bacterial leaf blight resistance, Blast resistance, Brown planthopper resistance, Linkage drag, Pseudo-backcrossing, Recurrent genome content, Donor genome content

## Abstract

**Background:**

Pyramiding multiple genes into a desirable genetic background can take years to accomplish. In this paper, a pseudo-backcrossing scheme was designed to shorten the backcrossing cycle needed. PinK3, an aromatic and potentially high-yielding rice variety—although one that is intolerant to flash flooding (Sub) and susceptible to bacterial leaf blight (BB), leaf-neck blast (BL) and the brown planthopper (BPH)—was used as a genetic basis for significant improvements through gene pyramiding.

**Results:**

Four resistance donors with five target genes (*Sub1A-C, xa5, Xa21, TPS* and *SSIIa*) and three QTLs (qBph3, qBL1 and qBL11) were backcrossed individually using markers into the pseudo-recurrent parent ‘PinK3’ via one cycle of backcrossing followed by two cycles of pseudo-backcrossing and three selfings with rigorous foreground marker-assisted selection. In total, 29 pseudo-backcross inbred lines (BILs) were developed. Genome composition was surveyed using 61 simple sequence repeats (SSRs), 35 of which were located on six carrier chromosomes, with the remainder located on six non-carrier chromosomes. The recurrent genome content (%RGC) and donor genome content (%DGC), which were based on the physical positions of BC_1_F_2_, ranged from 69.99 to 88.98% and 11.02 to 30.01%, respectively. For the pseudo-BC_3_F_3_BILs, the %RGC and %DGC ranged from 74.50 to 81.30% and 18.70 to 25.50%, respectively. These results indicated that without direct background selection, no further increases in %RGC were obtained during pseudo-backcrossing, whereas rigorous foreground marker-assisted selection tended to reduce linkage drag during pseudo-backcrossing. The evaluation of new traits in selected pseudo-BC_3_F_3_BILs indicated significant improvements in resistance to BB, BL, BPH and Sub compared with PinK3, as well as significant improvements in grain yield (21-68%) over the donors, although yield was 7-26% lower than in ‘PinK3’. All pyramided lines were aromatic and exhibited improved starch profiles, rendering them suitable for industrial food applications.

**Conclusions:**

Results show that our new pyramiding platform, which is based on marker-assisted pseudo-backcrossing, can fix five target genes and three QTLs into a high-yielding pseudo-recurrent background within seven breeding cycles in four years. This multiple pseudo-backcrossing platform decreases the time required to generate new rice varieties exhibiting complex, durable resistance to biotic and abiotic stresses in backgrounds with desirable qualities.

**Electronic supplementary material:**

The online version of this article (doi:10.1186/s12284-014-0035-0) contains supplementary material, which is available to authorized users.

## Background

Rice (*Oryza sativa* L.) is an important staple food crop and a major part of the diet of more than half of the world’s population. Approximately 90% of rice is grown in Asia. Worldwide, approximately 79 million ha of irrigated lowland rice provides 75% of the world’s rice production (Maclean et al. 2002; Bouman et al. 2007). Therefore, irrigated rice remains the most important production system for maintaining food security, particularly in Asian countries. Rice production in irrigated areas of Thailand has been frequently and strongly affected by abiotic stresses resulting from unfavorable climatic changes, such as flooding and drought, as well as by biotic stresses caused by bacterial leaf blight (BB), leaf/neck blast (BL) and brown planthopper (BPH). Therefore, new successful breeding lines must possess multiple types of resistance to both biotic and abiotic stresses, as well as demonstrating specific grain qualities and high yield.

Three popular breeding methods used for gene pyramiding are pedigree, backcrossing and recurrent selection. In cross-pollinating crops, gene pyramiding is accomplished through recurrent selection. Successful quantitative trait locus (QTL) introgression depends on the optimized expression of newly introgressed QTLs in the recipient genome background, with the aim of maximizing productivity. A general framework for addressing these considerations through the pyramiding of multiple QTLs into a single favorable genetic background has been proposed, although such techniques are time-consuming (Servin et al. 2004).

To pyramid several new QTLs, stepwise crossing schemes can be designed, although such schemes can require many generations of breeding. If the ultimate goal is to improve a specific desired variety, parallel backcrossing of single donors can most effectively be carried out using both foreground and background selection. Recently, successful gene/QTL pyramiding programs were reported in Thai Jasmine (Win et al. 2012; Luo and Yin, 2013), Basmati (Singh et al. 2012; Singh et al. 2013), Koshihikari (Ashikari and Matsuoka, 2006; Tomita, 2009), Zhenshan 97 (Wang et al. 2012) and 93–11 (Zong et al. 2012) rice varieties. However, when the target traits are quantitatively controlled, combining several QTLs can take years to accomplish. For multiple QTL pyramiding, three phases are necessary: the creation of near-isogenic lines (NILs), genotype assembly, and the extraction of pure lines. Specific backcrossed recombinant inbred lines (RILs) carrying four main-effect QTLs and four epistatic-effect QTLs were pyramided into the elite cultivar Zhenshan97 (Wang et al. 2012). To combine greater numbers of QTLs, marker-assisted phenotypic selection (MAS) has been developed, which is a novel approach for QTL pyramiding of up to 24 QTLs from a single crossing (Zong et al. 2012). QTL pyramiding via NILs was successfully used to improve disease and lodging resistance, as well as to increase the harvest index (Luo and Yin, 2013). However, all of these approaches require many years to complete.

To shorten the backcross breeding cycle, we propose a modified form of pseudo-backcrossing. The original design of pseudo-backcrossing originated from tree breeding methods, where F_1_ plants resulting from a single cross are backcrossed to alternate recurrent parents to avoid inbreeding depression (Bouquet 1986). Pseudo-backcrossing is commonly used in perennial plants, including grape (Molnár et al. 2007), eucalyptus (Kullan et al. 2012), poplar (Novaes et al. 2009) and oil palm (Montoya et al. 2014). For multiple gene/QTL pyramiding, several donors are used to create newly improved genotypes; however, the need to maintain the preferred genetic background is equally important. Therefore, the introduction of pseudo-backcrossing could benefit multiplex gene pyramiding.

Most rice varieties are not tolerant to flash flooding. Submergence tolerance is determined by the major Sub1QTL on chromosome 9 with relatively high heritability. The new Thai jasmine rice variety ‘Khao Dawk Mali 105 (KDML105)’ was developed for submergence (Sub) tolerance using marker-assisted backcross breeding (MAB) and has been released to farmers (Siangliw et al. 2003). Growing rice plants also suffer from epidemic diseases such as rice blast (BL) and bacterial leaf blight (BB). Rice BL, which is caused by the fungal pathogen *Pyricularia oryzae* (teleomorph: *Magnaporthe oryzae*), is a major rice disease in irrigated rice-growing areas worldwide (Ou 1985). QTLs for broad-spectrum resistance to rice BL have been reported on chromosomes 1 and 11 from JHN and on chromosomes 2 and 12 from IR64 (Sirithunya et al. 2002; Noenplab et al. 2006; Sreewongchai et al. 2010). Major effective QTLs for Thai blast isolates were located on chromosomes 1 and 11, which are flanked by RM212 and RM319 on qBL1 and by RM224-RM144 on qBL11 (Noenplab et al. 2006; Wongsaprom et al. 2010). The presence of qBL1 and qBL11 have strong effects on blast resistance in Thailand. BB, which is caused by the bacterium *Xanthomonas oryzae* pv oryzae can be effectively controlled using resistant varieties. Several resistance genes, including *Xa4, xa5* and *Xa21,* are effective sources of resistance for marker-assisted gene pyramiding in rice (Korinsak et al. 2009b; Suh et al. 2013).

Among insect rice pests, the brown planthopper (BPH, *Nilaparvata lugens* Sta° l), is considered one of the most serious pests of irrigated rice. The damage caused by BPH feeding has a major effect on crop growth and yield (Watanabe and Kitagawa, 2000; Yuan et al. 2005). BPH not only feeds on the rice plant directly but also transmits viruses that cause severe diseases (Heinrichs 1979). The use of BPH resistance genes has been recognized as the most economic, effective and environmentally friendly solution to this problem. The stability of BPH resistance in Rathu Heenati (RH), a traditional Sri Lankan rice cultivar containing qBph3, has made this strain one of the most popular hopper-resistance donors in the Mekong sub-region, where rice production is highly intensive. QTL mapping located qBph3 to the short arm of chromosome 6 between RM589 and RM588 based on KDML105 × Rathu Heenati (Jairin et al. 2009) and PTB33 × RD6 (Jairin et al. 2007) crossings. Other BPH resistance QTLs have also been reported, including Bph17 on chromosome 4 (Sun et al. 2005), Bph4 on chromosome 6 (Kawaguchi et al. 2001; Sun et al. 2006) and Bph18 on chromosome 12 (Jena et al. 2006). In addition to resistance QTLs, a putative sesquiterpene synthase (*TPS*) gene (Os04g27430**)** was identified using SFP mapping with isogenic lines derived from the backcross of RH and KDML105 (Kamolsukyunyong et al. 2013). The *TPS* gene is induced after 5 days of BPH feeding. Functional markers were identified in exon 5 of the *TPS* gene that resulted in the deletion of seven amino acids in the susceptible rice line, as well as three additional SNPs associated with a transcriptional binding site, accounting for the differential response of *TPS* during the anti-feeding test (Kamolsukyunyong et al. 2013).

Alkali disintegration has been used as a biomarker for gelatinization temperature (GT) in rice (Waters et al. 2006; Kate-ngam et al. 2008; Masouleh et al. 2012). The alkali disintegration locus (*ALK*) was identified as the *starch synthase IIa* (*SSIIa*) gene on chromosome 6 that determines amylopectin structure (Bao et al. 2004; Bao et al. 2006a; Umemoto et al. 2002; Umemoto et al. 2004; Umemoto and Aoki, 2005; Waters et al. 2006; Lu et al. 2010). Two functional SNPs were identified: GC/TT and G/A at positions 4329/4330 bp and 4198 bp, respectively (accession AY423717) (Bao et al. 2006b; Umemoto et al. 2002; Umemoto et al. 2004; Umemoto and Aoki, 2005; Waters et al. 2006; Lu et al. 2010). Successful marker assisted selection (MAS) programs utilizing the GT haplotypes for improving starch profiles have been reported (Tian et al. 2009; Lu et al. 2010).

‘PinK3’ is an aromatic, high-yielding, non-photoperiod-sensitive, high-amylose rice variety, but it is susceptible to BPH, BB, BL and Sub stresses. Here, we report the successful gene/QTL pyramiding of five functional genes (*xa5, Xa21, Sub1A-C, SSIIa, TPS*) and three QTLs (qBph3, qBL1, qBL11) into the ‘PinK3’ genome background using a multiplex pseudo-backcrossing approach based on MAS. The new, improved lines have a high-yield phenotype that confers submergence tolerance and resistance to BPH, BB and BL. This is the first report describing the application of pseudo-backcrossing to significantly shorten the time required for gene/QTL pyramiding in an annual crop (rice).

## Results

### Streamline gene pyramiding in rice

**Figure 1:**
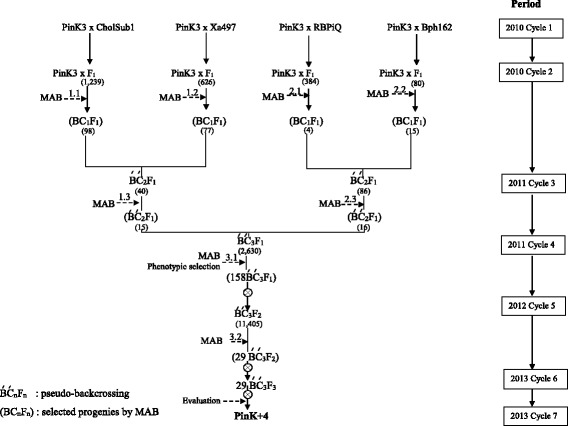
**The gene/QTL pyramiding scheme used to generate the high-yield pseudo-BC BIL‘PinK + 4’ line exhibiting submergence tolerance (** ***Sub1A-C*** **), bacterial leaf blight resistance (** ***Xa21*** **,** ***xa5*** **)** ***,*** **rice blast resistance (** ***qBL1*** **and** ***qBL11*** **), brown planthopper resistance (** ***qBph3*** **and** ***TPS*** **) and desired cooking qualities (** ***Wx, SSIIa, Os2AP*** **).**

Four sets of donors (Table 1) containing a set of target genes/QTLs—Cholsub (*Sub1A-C* and *SSIIa*), Xa497 (*xa5* and *Xa21*), Bph162 (qBph3 and *TPS*)*,* and RBPiQ (qBL1 and qBL11)—were backcrossed in parallel once to ‘PinK3’ by targeting MAB to generate four sets of BC_1_F_1_ progeny. The resulting BC_1_F_1_ progeny were stepwise crossed to recombine four sets of the target genes/QTLs into a single set of pseudo-backcrossed progenies. By pair-wise crossing two pairs of BC_1_F_1_ lines, two sets of pseudo-backcrossed progeny—BC_2_F_1_ (*Sub1A-C, SSIIa, xa5* and *Xa21*) and BC_2_F_1_ (qBph3*, TPS,* qBL1 and qBL11)—were generated (Figure 1). In cycle 4, all target genes/QTLs (*Sub1A-C, SSIIa, xa5, Xa21, TPS,* qBph3, qBL1 and qBL11) were recombined by crossing the two pseudo-backcrossed BC_2_F_1_ lines to generate 2,630 pseudo-backcrossed BC_3_F_1_ progeny. Using target MAS and plant-type selection, 158 fully heterozygous pseudo-backcrossed BC_3_F_1_ lines were selected and selfed (cycle 5) to generate 11,405 F_2_ progeny for large-scale, full-target MAS to generate 29 families for the target MAS purification. The numbers of positive plants (pseudo BC_3_F_2_) for all target genes/QTLs were segregated with Mendelian pattern (homozygous preference genotype = 1/4^n^). In cycle 6, selfing and full-target MAS yielded 29 best-selected, fully homozygous pseudo-backcrossed inbred lines (pseudo-BILs) carrying positive homozygous alleles of all of the donor genes, including *Sub1A-C, SSIIa, xa5, Xa21,* and *TPS* as well as qBph3 and qBL1-qBL11.

**Table 1:** **The four donors and recurrent parents used in the multiple backcross gene pyramiding**

**Cultivar/breeding line**	**Description**	**Variety type**	**Cross**	**Genotype on carrier chromosomes and target gene (s) or QTLs**											**References**
				***Sub***	***xa5***	***Xa21***	**qBL1**	**qBL11**	**Bph3**	***TPS***	***Wx***	***SSiia***	***Os2AP***	***Hd1***	
PinK3	High yielding aromatic rice (pseudo-recurrent parent)	RIL	IR71501/(KDMl105/CT9993)	-	-	-	-	-	+	-	+	-	+	+	Rice Science Center (unpublished)
CholSub1	Submergence tolerance aromatic rice with high GT	RIL	IR57514/KDMl105	+	-	-	-	-	-	-	-	+	+	+	Jantaboon et al. 2011
Xa497	BLB resistance aromatic rice	BC_2_F_2_	IR62266/KDMl105	-	+	+	-	-	-	-	-	-	+	+	Korinsak 2009
RBPiQ	Blast resistance	RIL	JHN/KDMl105	-	-	-	+	+	-	-	-	-	-	+	Rice Science Center (unpublished)
Bph162	Bph resistance photosensitive rice	BC_3_F_6_	Rathu Heenati/KDMl105	-	-	-	-	-	+	+	-	-	-	-	Jairin et al. 2009

+ = desirable allele.

- = undesirable allele.

Of these lines, five pseudo-BC_3_F_3_ BILs were chosen for field evaluation. The five pseudo BC_3_F_3_ BILs (PinK + 4) were selected based on completion of the target genomic regions with interesting starch profiles suitable for glycemic index research in the future (data not shown). In total, four donors and one recipient were intensively crossed and selected for seven cycles: two cycles to generate BC_1_F_1_, another two cycles to generate pseudo-backcrossed BC_3_F_1_, and three cycles of selfing to fix the final best-selected pseudo-BC_3_F_3_BIL for field evaluation.

### Graphical genotyping of pseudo-BIL

To determine the effects of pseudo-backcrossing on genomic background composition, nine elite pseudo-BIL (BC_3_F_2_) families were selected for genome scanning using 61 SSRs, 35 and 26 of which were located on six carrier and six non-carrier chromosomes, respectively (Additional file 1). The whole genome composition of the selected pseudo-BILs was characterized as the percentages of recurrent genome content (%RGC) and donor genome content (%DGC) based on the physical intervals of the SSR-based genome scanning using five BC_1_F_2_ (Table 2 and Additional file 2), two BC_2_F_2_ (data not shown) and nine pseudo-BC_3_F_3_BILs (Table 3 and Additional file 3). The %RGC and %DGC of BC_1_F_2_ ranged from 69.99 to 88.98% and from 11.02 to 30.01%, respectively (Additional file 2), whereas those variables for the pseudo-BC_3_F_3_BILs ranged from 74.50 to 81.30% and 18.70 to 25.50%, respectively (Additional file 3). The mean %RGC and %DGC values for BC_1_F_2_ were 80.04% and 19.96%, respectively (Table 2), whereas these values for the pseudo-BC_3_F_3_BILs were 77.48% and 22.52%, respectively (Table 3). These results indicated that at the BC_1_F_1_ step, there were no significant gains or losses of %RGC and %DGC from the two cycles of pseudo-backcrossing. For this reason, the theoretical RGC in pseudo-BC_3_F_3_ was not met.

**Table 2:** **The percentage of genome composition (average per target locus) of the selected BC**_**1**_ **F**_**2**_ **lines resulting from backcrossing of four donors: CholSub1, Xa497, RBPiQ, and Bph162 on PinK3**

	**% genome compositions (average per donor)**								
	**CholSub**	**Xa497**		**RBPiQ**		**qBph162**		**Ave.**	**Sum**
	***Sub1***	***xa5***	***Xa21***	**qBL1**	**qBL11**	***TPS***	**qBph3**		
Target gene/QTL	<0.01	<0.01	<0.01	0.03	0.26	<0.01	0.05	0.09	0.34
Donor segment link	1.81	0.00	3.77	1.59	0.00	0.00	0.00	1.79	7.17
Heterozygous segment link^1/^	0.00	0.83	0.13	0.12	0.95	0.00	1.16	0.80	3.19
Donor segment unlink	0.00	0.00	1.03	0.00	0.00	2.60	0.00	0.91	3.63
Heterozygous segment unlink^1/^	0.00	0.00	0.52	0.40	1.61	0.00	0.00	0.63	2.53
Sum of donor segments on target carrier chromosome	1.81	0.83	5.45	2.14	2.82	2.6	1.21	4.22	16.86
Donor segments on non-target carrier chromosome (4-5ch)								7.71	
^1/^Heterozygous segments on non-target carrier chromosome (4-5ch)								1.96	
Sum of donor segments on non-target carrier chromosome								13.89	
Donor segments on non-carrier chromosome (6 ch)								4.29	
^1/^Heterozygous segments on non-carrier chromosome								1.78	
Sum of donor segments on non-carrier chromosome								6.07	
Recurrent background								74.87	
**%RGC**								**80.04**	
**%DGC**								**19.96**	

^1/^= (1/2 total percentage of physical position).

**Table 3:** **The percentage genome compositions (average per target locus) in nine selected pseudo-BC**_**3**_ **F**_**3**_ **BILs**

**Region**	**% genome compositions (average per target)**						
	***Sub***	***xa5***	**qBL1**	***Xa21_*** **qBL11** ^***1/***^	***TPS***	**qBph3** ***_SSIIa*** ^***2/***^	**Sum**
Target gene/QTL	<0.01	<0.01	0.03	0.26	<0.01	0.05	0.34
Donor segment link	1.61	1.22	1.55	2.11	0.19	0.00	6.68
Heterozygous segment link	0.54	0.83	0.24	0.24	0.00	0.00	1.85
Donor segment unlink	0.00	0.18	1.39	1.40	1.44	0.47	4.88
Heterozygous segment unlink	0.00	0.00	0.04	0.23	0.00	0.76	1.03
Sum of donor segments on target carrier chromosome	2.15	2.23	3.25	4.24	1.63	1.28	14.78
Donor segments on non-carrier chromosome (6ch)							5.03
Heterozygous segments on non-carrier chromosome (6ch)							2.71
Sum of donor segments on non-carrier chromosome							7.74
Recurrent background							71.89
**% RGC**							**77.48**
**% DGC**							**22.52**

^1/^ *Xa21* located within qBL11 on chromosome 11.

^2/^ *SSIIa* located within qBph3 on chromosome 6.

We then looked into the distribution of donor genome segments across the six carrier and non-carrier chromosomes. For the carrier chromosomes, linkage drags were identified upstream and/or downstream of the donated target gene/QTL following transmission to the recipient genome. Even considering the stringent MAB on all target genes/QTLs during the BC_1_ cycle, linkage drags were still detected for almost every donated locus, both homozygous and heterozygous, on one or both sides of the target gene/QTL, constituting more than half of the total donor segments on the carrier chromosomes (Table 2). The largest linkage drags were detected on both sides of the *Xa21* locus (Table 2). However, only a small heterozygous linkage drag was detected at the *xa5* locus. The contrast between the degree of linkage drag for the two functional genes *Xa21* and *xa5* was unexpected, as the pair of loci was inherited from the same donor, Xa497. As both *xa5* and *Xa21* were MAB using their functional markers, it is interesting to speculate on the differences in linkage drag between the two functional genes. The size of the linkage of *Xa21* is much greater than for the two functional genes *Sub1* and *TPS*, and the other QTLs, qBL1, qBL11 and qBph3. Heterozygous linkage drags were identified for five of the eight target genes/QTLs, including the *Xa21* locus (Table 2), Donor-unlinks—the additional donor segments co-transmitted on the opposite (unlinked) chromosome arms of the target genes/QTLs—were identified on the carrier chromosomes containing *Xa21*, qBL1, qBL11 and *TPS* (Table 2). Therefore, the total donor segments transmitted via BC_1_ along with *Xa21* were obviously the largest among the target loci under MAB (Table 2).

Multiple target loci from donors are combined in the recipient genome, which include both linkage and non-linkage drags on the carrier chromosomes. Following two successive cycles of marker-assisted pseudo-backcrossing, BC_3_F_3_BILs linkage drags were detected innearly every case, with the exception of the qBph3-*SSIIa* locus. The total linkage drag (combined homozygous and heterozygous types) was 8.53%, which is more than half of the total donor component on carrier chromosomes (Table 3). The unlinkage drag values of the donated segments on the carrier chromosomes were between 5.91 and 40% of the total donor component on the carrier chromosomes (Table 3). To trace the potential origins of these large donor components, the sum of all donor compositions in the selected BC_1_F_2_ lines was compared with those components in the selected BC_3_F_3_BILs. On average, the donor genome content in BC_3_F_3_BILs was not significantly different from that of BC_1_F_2_ (Table 2 and Table 3). The same was true for the homozygous components of linkage drag and donor unlinked, whereas the heterozygous components were significantly decreased during pseudo-backcrossing. These results could indicate that high frequency of recombination between donated segments and the recipient genome contents of the carrier chromosomes in the BC_1_ cycle was primarily due to the high donor genome content in successive pseudo-backcrossings. Furthermore, the significant reduction in heterozygous donor components for the BC_3_F_3_BILs highlights an advantage of pseudo-backcrossing in gene pyramiding.

For the non-carrier chromosomes, the total donor components on all six non-carrier chromosomes were not significantly affected (Table 2 and Table 3). However, the heterozygous donor components on both carrier and non-carrier chromosomes persisted, even in the selected pseudo-backcrossed BC_3_F_3_BILs. These results show that pseudo-backcrossing has similar effects as conventional backcrossing when considering the recovery of the genome content on non-carrier chromosomes.

### Evaluation of the PinK + 4 phenotype

Field evaluations of agronomic and grain quality were performed based on complete target genes/QTLs, plant types, days to harvest and grain quality. The foreground selection successfully fixed homozygosity of the five target genes (*Sub1A-C, SSIIa, xa5, Xa21* and *TPS*) and three QTLs for BL and BPH into approximately 77% of the genetic background of the pseudo-recurrent parent ‘PinK3’ (Table 3 and Additional file 3). However, the majority of the advanced progeny exhibited significant phenotypic variation from their pseudo-recurrent parent ‘PinK3’ with respect to nearly all evaluated traits, with the exception of amylose content, grain length per width ratio and polished grain length (Tables 4 and 5).

**Table 4:** **Trait evaluation and agronomic characteristics of four selected pseudo-BC**_**3**_ **F**_**3**_ **BILs (PinK + 4) and parental lines with respect to submergence, bacterial blight, brown planthopper and blast resistance**

**No**	**Name**	**Family**	**Trait evaluation** ^**1/**^				**Agronomic characteristics** ^**2/**^						
			**Sub**	**BLB (TXO156)**	**Bph(UBN)**	**Blast (Mixed#2)**	**DM**	**NTP**	**PH (cm)**	**NGP**	**PSF (%)**	**TGW (g)**	**GY (kg/ha)**
1	1E_06	1_H06	50.7^c3/^	1.5^a^	1.7^a^	0.7^a^	140.0^e^	9.3^ab^	70.1^bc^	276.7^e^	82.5^ef^	42.9^f^	7777^ef^
2	20A09	4_E02	42.0^bc^	1.4^a^	1.7^a^	0.7^a^	140.0^e^	9.3^ab^	79.0^c^	224.3^d^	69.6^c^	41.4^e^	7381^ef^
3	66B09	3_E03	31.3^b^	0.8^a^	1.7^a^	0.6^a^	128.0^c^	9.2^ab^	77.7^c^	236.1^d^	76.1^d^	42.5^f^	6977 ^de^
4	78A03	13_H06	52.0^c^	0.8^a^	3.7^abc^	0.0^a^	143.0^h^	9.2^ab^	78.3^c^	241.0^d^	77.3^d^	42.4^f^	6966^de^
5	117A08	2_A10	56.0^c^	1.5^a^	3.0^ab^	0.0^a^	126.0^c^	8.6^a^	77.3^c^	168.7^c^	81.1^e^	41.5^e^	6175^cd^
6	PinK3	recurrent	0.0^a^	12.8^b^	6.3^cd^	5.7^d^	141.3^f^	8.3^a^	77.0^c^	301.0^f^	62.0^b^	40.3^d^	8306^f^
7	Xa497	donor	3.3^a^	0.5^a^	6.3^cd^	3.7^c^	134.0^d^	9.5^ab^	59.3^a^	166.0^c^	84.9^f^	40.5^d^	5417^c^
8	CholSub1	donor	50.7^c^	nd	5.7^bc^	2.3^b^	122.0^b^	10.7^bc^	62.7^ab^	120.3^b^	75.0^d^	39.4^c^	4041^b^
9	RBPiQ	donor	2.0^a^	18.4^b^	9.0^d^	0.0^a^	142.0^g^	10.4^cd^	74.3^c^	185.7^c^	94.1^g^	34.8^a^	5581^c^
10	Bph162	donor	0.0^a^	15.6^b^	1.0^a^	3.3^c^	99.0^a^	12.8^d^	69.7^bc^	81.0^a^	58.5^a^	38.5^b^	2227^a^

Remarks:

^1/^Trait evaluation.

Sub = Average % plant survival (%PS) after 15 days of flash flooding.

BLB = average lesion length in centimeters of the damage caused by the BB isolate TXO156.

Bph = Severity scores with UBN biotype at 9 DAI when TN1, the susceptible control died.

Blast = Average blast injury score when attacked by a Mixed#2 blast isolate from Thailand.

^2/^Agronomic characteristics.

DM (days to maturity), NTP (number of tillers per plant), PH (plant height from the soil surface to the neck of the panicle), NGP (number of grains per panicle), PSF (percent spikelet fertility), TGW (1,000 grain weight) and GY (grain yield).

^3/^Average values marked with different letters in the same column are significantly different at the 95% confidence level using LSD.

**Table 5:** **Grain quality and cooking quality traits of five pseudo-BC**_**3**_ **F**_**3**_ **BILs (PinK + 4) as well as the donor and recurrent parents**

**No.**	**Name**	**BR** ^**1/**^ **(%)**	**HR** ^**1/**^ **(%)**	**GL/W** ^**1/**^	**PRL** ^**1/**^ **(mm)**	**AC** ^**1/**^ **(%)**	**ASV** ^**1/**^	**CE** ^**1/**^ **(%)**
1	1E_06	72.9^c2^	51.8^c^	3.0^ns^	0.75^ns^	29.3^c^	7.0^c^	36.7^abc^
2	20A09	72.9^c^	52.7^c^	3.1^ns^	0.74^ns^	27.6^c^	7.0^c^	43.3^cdef^
3	66B09	71.5^b^	52.1^c^	3.1^ns^	0.76^ns^	27.5^c^	2.0^a^	31.5^a^
4	78A03	71.2^b^	51.3^c^	3.2^ns^	0.78^ns^	27.1^c^	2.0^a^	31.2^a^
5	117A08	73.0^c^	53.1^c^	2.9^ns^	0.73^ns^	29.5^c^	7.0^c^	46.7^ef^
6	PinK3	72.4b^c^	43.4^b^	3.1^ns^	0.74^ns^	29.2^c^	7.0^c^	39.4^bcd^
7	Xa497	71.9b^c^	53.3^c^	3.4^ns^	0.74^ns^	22.0^b^	5.0^b^	40.3^bcde^
8	CholSub	73.2^c^	42.4^b^	3.4^ns^	0.74^ns^	14.5^a^	2.0^a^	44.4^def^
9	RBPiQ	71.0^b^	41.3^b^	3.4^ns^	0.68^ns^	14.2^a^	5.0^b^	35.9^ab^
10	Bph162	68.6^a^	30.2^a^	3.2^ns^	0.70^ns^	14.0^a^	5.0^b^	48.6^f^

Remarks: ^1/^BR (brown rice), HR (head rice), GL/W (grain length-width ratio), PRL (polished rice length), AC (amylose content), ASV (alkaline spreading value (1.7% KOH)), and CE (cooking elongation).

^2/^Average values marked with different letters in the same column are significantly different at the 95% confidence level using LSD.

Rice grains were harvested from the yield trial field during the wet season of 2012/2013.

**Figure 2:**
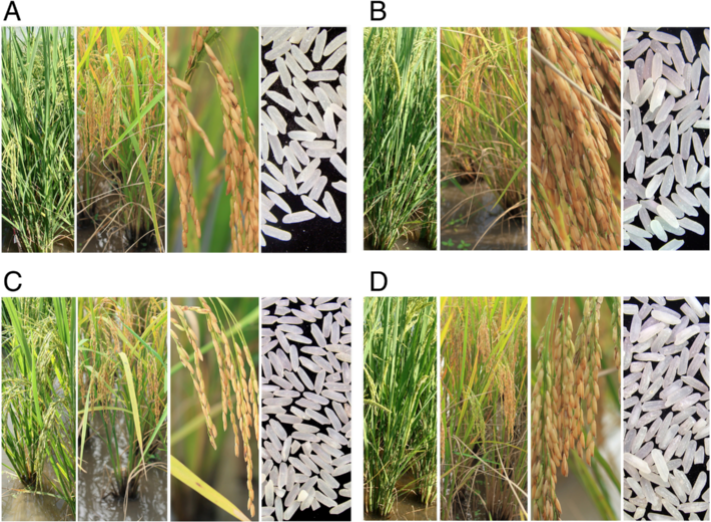
**Plant and grain types of pseudo-BC BIL PinK + 4 compared with PinK3 (pseudo-recurrent parent). A)** PinK + 4#1E06, **B)** PinK + 4#20A09, **C)** PinK + 4#66B09 and **D)** PinK3.

The uniformity of the pseudo-BC_3_F_3_BILs was the result of MAS. Even with respect to complex traits, such as grain yield, some of these progeny performed as well as the pseudo-recurrent parent ‘PinK3’ and significantly outperformed their donors (Table 4 and Figure 2). However, some progeny inherited inferior characteristics from the resistance donors, which affected maturity, grain numbers per panicle (NGP), % seed fertility (PSF) and grain yield (GY). In all cases, early-maturing progeny produced a lower grain number per panicle and lower grain yield (Table 4). Therefore, without rigorous background selection, pseudo-backcrossed progeny may not possess the desirable characteristics of their pseudo-recurrent parent.

For the *Sub1* selection, all progeny exhibited a significantly improved ability to withstand flash flooding during seedling stages compared with the susceptible ‘PinK3’ (Table 4). However, significant phenotypic variation (31–56%) was observed among the Sub1-Pink3 families compared with the control phenotype. Two reasons for this variation; First, the *Sub1* gene was not directly inherited from the original FR13A but from CholSub1 donor one of the RILs from the mapping pop ‘KD × FR13A’ (IR57514) for submergence tolerance QTL by Siangliw et al. (2003). The second reason is the quantitative nature of multiple gene/QTL may partially regulated by recipient genetic background of CholSub1 donor and the ‘PinK3’, the highly susceptible recipient. Furthermore, the fact that significant variations in traits related to submergence tolerance were observed among the individual progeny of IR57514/Kao Dawk Mali 105 (Jantaboon et al. 2011) is also consistent with our findings, indicating the quantitative nature of such traits.

For BB, selection based on *xa5* and *Xa21* resulted in significant improvements in bacterial leaf blight resistance, specifically to the TXO156 virulent isolate (Table 4) and to several BB isolates identified in Thailand (Additional file 4). For leaf/neck blast resistance, QTL pyramiding of the two QTLs on chromosomes 1 and 11 onto the ‘PinK3’ background successfully improved resistance to a wide range of blast isolates collected in Thailand (Table 4).

For BPH resistance, based on the UBN biotype (a BPH biotype that has been well characterized in Thailand), the progeny exhibited significant improvement over their pseudo-recurrent parent ‘PinK3’ but were not as resistant as the resistance donor. The Bph3 QTL and the *TPS* gene from the Bph162 donor were co-inherited from the broad spectrum BPH resistance cultivar ‘Rathu Heenati’ in crosses with KDML105, as reported by Jairin et al. (2009). Some selected introgression lines from that report were moderately resistant (MR) to various BPH biotypes. It appears that introgression of only qBph3 and *TPS* from Bph162 in pseudo-backcrossed BILs was not enough to withstand some of the BPH biotypes used in our experiments.

All selected pseudo-BC_3_F_3_ BILs (PinK + 4) contained the aromatic allele and the *Wx*^*a*^ allele known to confer high amylose content and a starch profile suitable for further analysis and industrial food applications.

## Discussion

### Streamline backcrossing design

In a gene-pyramiding project, several donors, each providing a target QTL with flanking markers, are used as parental sources for new traits with the goal of improving a favorable variety that has a preferred genetic background. To best facilitate the efficient integration of multiple QTLs into a single optimal variety, an improved breeding platform was developed based on pseudo-backcrossing. In conventional backcross breeding experiments, nearly isogenic lines from each donor were developed prior to pyramiding (Luo and Yin, 2013; Singh et al. 2013) to recover the background genome of the recurrent parent. However, when introducing multiple traits, this approach can be tedious and time-consuming. This novel platform based on pseudo-backcross breeding involves both multiple foreground selection and background genome recovery in an abridged manner. Within seven cycles—consisting of a single backcross, two cycles of pseudo-backcrossing and three cycles of line fixation—the entire project was accomplished within four years.

### Recurrent genome background recovery

Transferring multiple resistance genes using conventional MAB requires at least three to four backcrosses to guarantee a high recovery of the recurrent parent phenotype (Joshi and Nayak, 2010; Suh et al. 2013). In this study, without background selection, the pseudo-BC_3_F_3_BILs (PinK + 4) contained 74.50 to 81.30%RGC, which is significantly below the theoretical value of 93.75% possible following three conventional backcrossings. When comparing graphical genotyping among BC_1_F_2_ progeny and pseudo-BC_3_F_3_BILs, the total %DGC was only slightly increased. These results indicate that without background selection, pseudo-backcrossing can only maintain the %RGC gained during the first backcross generation. A low background recovery rate was also reported for the introgression of stripe rust resistance in wheat. Without marker-assisted background selection, the %RGC was only 82% in BC_4_F_7_ progeny (Randhawa et al. 2009). However, when combined with phenotypic selection, %RGC was improved to 85–92% in BC_3_ (Sundaram et al. 2008; Korinsak et al. 2011; Singh et al. 2013). Indeed, the utilization of genome-wide molecular markers for background screening during backcrossing has been suggested as the best method for improving low %RGC (Rajpurohit et al. 2011; Suh et al. 2013).

The number of molecular markers used for genome-wide scanning, which ultimately determines cost vs. precision, has varied from 44 to 205 SSR loci in rice. Four groups have reported rice MAB projects involving background selection: Group 1) used 44–51 SSRs (Yi et al. 2009; Siangliw et al. 2003; Tomita 2009; Wongsaprom et al. 2010), Group 2 used 67–72 SSRs (Win et al. 2012; Singh et al. 2013), Group 3 used 84–97 SSRs (Siangliw et al. 2007; Jantaboon et al. 2011), and Group 4 used 107–205 SSRs (Rajpurohit et al. 2011; Korinsak et al. 2011; Suh et al. 2013). However, the majority of these MAB projects utilized molecular markers during the final stage of selection.

### Linkage drag

In conventional backcrossing, many portions of the donor genomes are inserted into both the carrier and non-carrier recipient chromosomes during early cycles. After continued backcrossing, the donor genome segments are gradually replaced by sequences from the recurrent parent at varying rates. In most backcrossing programs, linkage drag is responsible for long-lasting donor genome segments remaining in the recurrent genome. However, there is no difference in terms of linkage drag in pseudo-backcrossing schemes. In pseudo-backcrossing BILs, there was only a 1% reduction in linkage drag from BC_1_ to pseudo-BC_3_, and most of this reduction was due to the heterozygous segments of the linkage drag. Our results also revealed that the degree of linkage drag is independent of the size of the target genes/QTLs. When comparisons were made between the selection of single genes or single QTLs, the degree of linkage drag was less for QTLs than for single genes. Of the five single gene selections, *SSIIa, Sub1, xa5, Xa21* and *TPS*, only selection for *Xa21* showed large, persistent linkage drag. Persistent linkage drags when selecting for BB genes have been reported in several backcross breeding programs, such as during the transfer of *Xa4 + xa5 + Xa21* from indica ‘IRBB57’ into japonica ‘Mangeumbyeo’ (Suh et al. 2013), and pyramiding of the BB resistance genes *Xa21* and *xa13* and a semi-dwarfing gene (*sd-1*) from PR106-P2 into Type 3 Basmati (Rajpurohit et al. 2011). The problem of persistent linkage drag when selecting for *Xa21* may due to the fact that *Xa21* was derived from IRBB21, which inherited the chromosomal region containing *Xa21* from the wild species *Oryza longistaminata* through several cycles of backcrossing with indica rice (Song et al. 1995). The degree of linkage drag may depend on linkage disequilibrium surrounding the target gene to be transferred. Genes inherited from wild species in cultivated strains may be retained within long, stable LD stretches that are difficult recombine. Therefore, selection based on functional markers alone does not guarantee linkage drag-free progeny. More successful single-gene target selection has been reported when markers flanking the gene of interest were also selected for (Rajpurohit et al. 2011). In one of the most comprehensive backcrossing projects, a single gene, *sd1,* was integrated into the desirable variety ‘Koshihikari’ using eight cycles of MAB with 51 SSR markers surrounding *sd1* to completely eliminate linkage drag (Tomita 2009).

### Grain yield performance of pseudo-BC BILs

In this study, we combined multiple resistance genes from four donors using a new backcrossing method involving pseudo-backcrossing. The results show that all pseudo-BC BILs showed significant improvements in resistance to BB, BL, BPH and Sub compared with the pseudo-recurrent parent ‘PinK3’, as well as significant improvements in grain yield (21–68% over the donors, but 7–26% lower than the recipient). The reduction in grain yield in the pseudo-BC BILs should be interpreted in several ways. First, there was an average of 7.6% and 22.5% of linkage drag and DGC, respectively, in the recurrent genome. As these donors were inferior in grain yield with respect to the pseudo-recurrent parent ‘PinK3’, the high persistent %DGC could have disrupted the optimal expression of high-yield genes in pseudo-BC BILs. Second, there can be a slow recovery of the %RGC during pseudo-backcrossing when MAS for the recurrent background is not in place. Under such conditions, reconstruction of the recurrent genome content by recombining different pseudo-backcrossed lines is not favorable, as different donor segments on both carrier and non-carrier chromosomes have more chances to recombine, creating new substitution lines that do not resemble the recurrent parent. Third, the multiple donated genes/QTLs from donors to the recipient act as a ‘genetic load’ against the fitness of the recurrent parent. The over-expression of multiple resistance genes could counteract the metabolic energy needs necessary for high yield. Therefore, pseudo-backcrossing may be the fastest method for gene/QTL pyramiding, although it may not be the ideal breeding platform for creating elite recurrent varieties. However, marker-assisted, genome-wide scanning can be implemented during early stages to facilitate the reconstruction of favorable genomic backgrounds at the end of the pseudo-backcrossing scheme. Ultimately, the trade-offs must be considered by the breeders. Ideally, new high-throughput, low-cost, genome-wide scanning technologies should be utilized in combination with skillful breeder selection. For the whole project, more than 50,000 plants were individually genotyped for at least one molecular marker. If budget is allow, extensive background selection must be emphasized.

## Conclusion

We improved high-yield, aromatic rice varieties by introducing desirable multiple traits by pyramiding five target genes (*Sub1A-C, SSIIa*, *xa5, Xa21* and *TPS*) and three QTLs (qBph3*,* qBL1 and qBL11) from four resistance donors. We redesigned the gene-pyramiding platform to minimize the total project time span by integrating MAS into pseudo-backcross breeding. Consequently, only seven breeding cycles in four years were required to develop new varieties exhibiting multiple resistance traits. Using pseudo-backcrossing, approximately 77.48% of the recurrent genome background was recovered. With additional background genome selection, the recurrent genome background can further improve the %RGC and optimize the expression of introgressed QTLs.

## Methods

### Plant materials used

PinK3 is a high-yield, irrigated aromatic rice cultivar developed by Rice Science Center, Kasetsart University, Thailand (unpublished). However, this variety is susceptible to flash flooding (Sub), bacterial leaf blight (BB), leaf-neck blast (BL) and the brown planthopper (BPH). The four donors used to transfer five genes and three QTLs to the pseudo-recurrent parent ‘PinK3’ (Table 1) were developed by the Rice Gene Discovery and Rice Science Center, Kasetsart University, Thailand. The four donors used to improve the abiotic and biotic stress tolerances of ‘PinK3’ are listed in Table 1.

### Pseudo-backcross design

The pseudo-backcross platform is divided into three steps. In the initial step, one round of backcrossing is conducted to donate the favorable QTL allele to the recipient background using marker-assisted backcrossing (MAB). Each QTL-BC_1_F_1_ contains approximately 75% of its recurrent genome content (RGC). In the second step, the BC_1_F_1_-plus QTLs are used as pseudo-recurrent parents, and pseudo-BC_2_F_1_ plants are formulated by crossing between them. More BC_1_F_1_ plus new QTLs can be crossed to successively generate pseudo-BC_n_F_1_ QTLs and, thus, to continue streamline gene pyramiding. At the end of this step, the BC_n_F_1_, which contains the new QTLs at full heterozygosity at all target marker loci, are self-pollinated to fix the target loci (Additional file 5). In rice, this new platform allows breeders to stack more QTLs in the shortest possible time (shorter than that required by any other method).

### Donors for gene pyramiding in rice

Four donors providing submergence tolerance, bacterial leaf blight resistance, blast resistance, BPH resistance and desired gelatinization temperature were introduced into PinK3 (*aroaro* and *Wx*^*A*^*Wx*^*A*^) as the female pseudo-recurrent parent. The donor CholSub1 carries two target traits: submergence tolerance and desired gelatinization temperature (*Sub1A-C* and *SSIIa*); the donor Xa497 carries two functional genes for bacterial leaf blight resistance (*xa5* and *Xa21*); the donor RBPiQ carries two QTLs for blast resistance (qBL1 and qBL11); and the donor Bph162 carries two target traits for BPH resistance (qBph3 and *TPS*) (Table 1). These four donors were used for gene pyramiding.

### Genomic DNA isolation

Rice seedlings from each segregating population were grown in a 288-well plastic tray (representing three 96-well plates). Young leaves from 14-day-old individual plants were cut into small pieces and placed (~0.2 g weight per sample) in a 2-ml 96-well plastic block. Leaf tissues were ground in liquid N_2_ using a Tissue Striker II (KisanBio, Seoul, South Korea). After grinding, 300 μl Agencourt®Chloropure lysis buffer was added to the samples. Homogenized tissues were incubated in a 2-ml 96-well plastic block at 65°C for 1 hour. The sample blocks were then centrifuged at 4,000 rpm for 10 minutes. Lysates (containing at least 200 μl) were transferred to a new 2-ml 96-well plastic block using an Automated Biomeck NX AP96 instrument (Beckman Coulter, California, USA). The extraction was conducted using the standard protocol of Agencourt Chloropure for nucleic acid isolation from plants (Beckman Coulter, California, USA).

### Foreground MAS

Foreground selection was performed using two marker systems. For SNP and functional markers, multiplex genotyping was conducted using the SNPstream system (Beckman Coulter, California, USA). The remainder of the foreground markers were SSR markers flanking specific QTLs. The SNP-based genotyping array was described.

### High-throughput multiplex SNP genotyping

High-throughput genotyping was performed by multiplex PCR, as described by Bell et al. (2002) with certain modifications. In brief, the forward/reverse (18–20 nt in length) and SNP-specific (40–45 nt in length) primers were designed for each foreground locus (Primers can be manually designed) to generate a product of 90–180 nt in size. The program selects the best Single Base Extension (SBE)-primer based on sequence melting temperature (T_m_; °C) and secondary structure. At the 5′ end of the SBE-primer sequences are 20-nt tags that are complementary to the sequences of specific positional tags in the SNPware (384-well) microarray format (Beckman Coulter, California, USA) (Table 6).

**Table 6:** **Primer sequences used in the SNP genotyping format for foreground selection**

**No.**	**SNP name**	**Chr.**	**Trait**	**Tag number**	**SNP type**	**Amplicon size (bp)**	**Forward primer (5′---3′)**	**Reverse primer (5′---3′)**	**SNP-specific primer (5′---3′)**
1	Sub1C	9	Sub	9	A/G	111	ACGAGCCGACGACGACGA	ATCTCCGACGCCCACCTC	CCGCCAGTAAGACCTAGACGCGGCGGCGGCGGCGGCGGAGGGAGA
2	xa5*	5	BB	2	C/G	303	GGCCACCTTCGAGCTCTACC	CAACATTGCAACTCCGTGATAAG	CTCAGACTACGAATCCACGTGTAAAGTAGATACCTTATCAAACTG
3	Xa21	11	BLQ11	40	T/C	141	AAAGCTAGGCTGCTAGTGCTG	AAAATAGTATATATGTACCACTGCTTCTT	AAGTACCACGTCAACGTCACTATGCTTCAAGGTCAGGGTGGTCGA
4	SSIIa	6	GT	25	G/T	89	CCACTGCCTCGAGACGTA	CGTGGTCCCAGCTGAGGT	CCATAACAACTTACCAGCCAGCAAGTACAAGGAGAGCTGGAGGGG
5	Waxy	6	AC	31	G/T	141	TTCACTTCTCTGCTTGTGTTGT	TACTTGTAAGGAAAAACGAGCAA	CAGAACATCCTCAGAAGCAAGTTCATCAGGAAGAACATCTGCAAG
6	aromarker*	8	FR	41	A/G	400	AATCATGTATACCCCATCAA	TTTCCACCAAGTTCCAGTGA	TACCTATGACCAGCAAGCACAACCTTAACCATAGGAGCAGCTGAA
7	Hd1	6	Photo	13	C/G	152	TCCAAAGATTCCGACAACA	TTGTCGTAGTACGAATTGTACCC	CAACAATACGAGCCAGCAAGACAACAACAACGACAACGACAATAA
8	TPS_Chr4_ATHB1*	4	Bph3	27	T/G	264	AAGCGCTTATATTCAAGCAGAA	TCCATTCTTCCGATCTCTGG	GCAAGCCATCAGCTAATACATTCATGAAACAGTTCTAGCAATAAT
9	Bph_Chr6_1210*	6	Bph3	17	T/C	287	GAAAGCCTTTGAAACAAAGTATTGA	CTTGAATTTGAAGTTGATTTTAGGG	GCAGACAACGAACAACTACCAAACGGCATATTTGCAAACAGAAAA
10	Bph_Chr6_3380*	6	Bph3	22	T/C	300	AGAGGAAATGATTCAAGGAG	AGCTAGCAGGCGTAGCTTAT	GCAACATAAGACCGCTCAACTAGTTAATTTCACGCCATGACAGAT
11	Bph_Chr6_1380*	6	Bph3	19	A/G	296	TTTTGTTTTCTTCTTGAGAGTGGTC	CAAGGTAATGACATCAAGAACCAAT	CCACTCAACTCCACGAATACCACATGTTTATTTTTAATTTCACAG
12	TBGI055716	1	BLQ1	24	T/A	169	ACGATGCGGCACTCGTCG	TGTTCTTGAACGCGGCGA	CAACAAGACATAACAACGCAGTGGAGTGGTGGATGAAGCGGAAGA
13	TBGI055578	1	BLQ1	42	A/G	93	ATTTGCTGCTCATGGTGG	TGGGGAAGCCGAGGAGAT	CTCACTATCTGACAAGCCACGTGGAGCCTCCTCACCAGGAAGTGC
14	TBGI055841	1	BLQ1	46	T/C	115	ATTGGCATCGTTTGGTCTG	ATTCCGTGCATATATACGAACTTC	ACAGATCACTCACCGACTAAATACCTGCGTCGAGTAGAGACGATG
15	TBGI454069	11	BLQ11	14	A/G	142	TGAATTGTCGTCCTCTAACAAC	TATGGAATATCTGCATCATGAAC	AACATACAGACGCACTCCTCTCTTTGCTATGCCAAAGTCTGCTAC
16	TBGI453598	11	BLQ11	23	C/T	90	TTTGCTGTGACGGGAAGA	AAAAAGGAACTAGCCAGTTTTG	AGTAGCCTAACAGCACTCGAAAGATCGAGTGCTCTATTGCAACCG
17	TBGI453126	11	BLQ11	32	C/T	92	ACCGACGCTGCTGCAGAA	AGCGGTTATGGATGGCTAA	CAAGCAACGACCTACTACAAGCATGGCGTTTGAGCGCGTCCTGGG
18	TBGI454717	11	BLQ11	36	T/A	132	ATCCTACCGTCCGCTCTG	AATTCGGTCTTCGTAAACACG	CACCGCTATCAACAGACTTGGTGCGGTAGTTTCTGGGAAGCTACG
19	TBGI454800	11	BLQ11	38	G/C	113	TACTACAACAACAGGAACGCC	TTGATGATGAAGTGGATGAGC	ACGTAAGACCACTCAAGACCAGAAGACGCTGAACAGGATGGCGAT

*These primer designs were optimized manually.

### Multiplex PCR

A 10-μl PCR reaction containing 5 μl genomic DNA (10 ng/μl) and 5 μl KAPA TaqHotStart PCR buffer (1 U KAPA TaqHotStart (KapaBiosystems, MA, USA) (final concentration of 1× KAPA TaqHotStart Buffer: 75 μM dNTPs, 5 mM MgCl_2_ and 50 nM 38-primer pool) was performed in a 384-well PCR plate (Sorenson BioScience, UT, USA). The following thermocycler touch-up PCR cycle was used: 95°C for 3 min, followed by 6 cycles of 95°C for 30 seconds, 50°C up to 55°C (0.3 increment/cycle) for 30 seconds, and 72°C for 30 seconds; this was followed by 34 cycles of 95°C for 30 seconds, 55°C for 30 seconds, 72°C for 30 seconds and a final extension at 72°C for 7 min. Subsequently, the temperature was held at 4°C.

Multiplex PCR assays were prepared separately based on the SNP panel type (A/G, A/C, A/T, G/C, G/T and C/T). Following PCR amplification, PCR products were cleaned, and the SBE reactions were performed. Next, multiplex SBE products from different panel types were pooled prior to the hybridization step.

PCR clean up, SBE reactions, hybridization and washing, SNPstream imaging and data analysis were performed as described by Bell et al. (2002).

### Evaluation of abiotic and biotic stress traits

#### Submergence screening

The parents and pseudo-BC_3_F_3_BILs (PinK + 4) were screened for submergence resistance traits. The experiment was conducted under complete submergence in an outdoor lagoon located at the Rice Science Center, Kasetsart University, Kamphaeng Sean Campus, Thailand, during the dry season of 2013. The experiment was arranged using a randomized complete block design with three replications. Sixteen three-week-old BC_3_F_3_BIL seedlings and controls including PinK3, CholSub, Xa497, RBPiQ and Bph162 were transplanted in three replicate plots (plot size: 0.75 × 0.75 m^2^) at a spacing of 25 cm × 25 cm. Two weeks after transplanting, the number of seedlings was counted in each plot; then, the lagoon was filled with water to a depth of 2 m. To impose severe submergence stress, the seedlings were completely submerged for 15 days; the water level was maintained at 1–1.2 m above the leaf tip throughout the experimental period. After this period, the lagoon was drained, and the seedlings were re-exposed to air for 10 days (Jantaboon et al. 2011). The number of surviving plants was recorded. The percentage of survival (PS) was calculated using the following equation:

$$ \mathrm{P}\mathrm{S}=\frac{\mathrm{Number}\kern0.35em \mathrm{of}\kern0.35em \mathrm{surviving}\kern0.35em \mathrm{plants}}{\mathrm{Total}\kern0.35em \mathrm{number}\kern0.35em \mathrm{of}\kern0.35em \mathrm{plants}}\times 100 $$

#### Brown planthopper (BPH) screening

A set of pseudo-BC_3_F_3_BILs (PinK + 4) and their parents were screened for resistance against BPH using standard seedbox screening (SSBS); the BPH population used was collected from Ubon Ratchathani provinces (Jairin et al. 2007). The SSBS was conducted at the seedling stage (10 days old) under greenhouse conditions following the method described by Heinrichs et al. (1985). Damage scores were recorded when the susceptible control, ‘TN1’, died (9 days after infestation; 9DAI), using the standard evaluation system (IRRI 1996).

#### Bacterial leaf blight screening

The *Xoo* isolate TXO156 was selected for this experiment. The isolate was grown following the method described by Win et al. (2012). The parents and pseudo-BC_3_F_3_BILs (PinK + 4) were grown in a greenhouse for 30 days before inoculation. The inoculation procedures used were adapted from those described by Korinsak et al. (2009a, b). Three to four fully expanded leaves of each plant were inoculated. Lesion length (LL) was measured at 12–14 days after inoculation. The resistance reaction was classified as resistant (R), moderately resistant (MR), moderately susceptible (MS) and susceptible (S) when the values of LL were 0–3 cm, 3.1–6 cm, 6.1–9 cm and more than 9 cm, respectively (Yang et al. 2003; Lin et al. 1996).

#### Leaf blast screening

Thailand *Magnaporthe oryzae* mixed isolates#2, including THL710 (Mae Hong Son), THL282 (Phrae), THL906 (Yala), THL122 (Chiang Rai), THL757 (Mae Hong Son) and THL603 (Surin) (Rice Gene Discovery, Thailand, unpublished), which can damage the PinK3 form in the mixed isolate pre-screening, was used in leaf blast screening experiments. The inoculum was prepared and the plants were inoculated following the method described by Marchetti et al. (1987) with some modifications. Pseudo-BC_3_F_3_BILs (PinK + 4) and their parents were grown in polyvinyl trays containing paddy field soil (four seedlings per line) following a three-replication completely randomized design (CRD). The seedlings were maintained in a greenhouse for 17 days before inoculation, after which they were inoculated with mixed isolates#2. Disease scoring was recorded at seven days after inoculation on a 0 to 6 scale following the procedure described by Roumen et al. (1997) and IRRI (2002). The average score of each line was computed from the disease score measured for 12 individual plants.

### Recording of important agronomic traits

Traits measured included days to 100% flowering (DF100), days to maturity (DM), number of tillers per plant (NTP), plant height (PH), number of grains per panicle (NGP), percent spikelet fertility (PSF), 1,000 grain weight (TGW) and grain yield (GY); these traits were measured for rice plants grown during field trials at Kasetsart University, Kamphaeng Sean, Nakhon Pathom, Thailand.

Twenty-one-day-old seedlings were transplanted in three replicates in 1 × 2 m^2^ plots using 25 × 25 cm^2^ plant spacing. Agronomic traits were recorded for five randomly selected plants grown in each plot. The DF was recorded when 100% of the individual plants in each plot flowered. NTP, PH, NGP and DM were measured at maturity, and the results were averaged from five randomly selected plants in each plot. PH was measured from the soil surface to the neck of the panicle. The NGP was counted manually for five panicles. To measure the GY in each plot, only the inner rows (containing 21 plants) were used. Two border rows on each side of the plot and the border plants of each row were discarded. The GY recorded for each plot was adjusted to 14% moisture content and then extrapolated to units of kg per ha. TGW measurements were replicated three times. Statistical analysis was performed using the STATGRAPHICS plus 3.0 software package (Manugistics 1997).

### Evaluation of grain quality

Grain quality was evaluated using grain harvested from the trials field. Rice grains of the pseudo-BC_3_F_3_BILs (PinK + 4) and their parents were harvested at physiological maturity and sun-dried in a greenhouse. The dried grains were stored at room temperature for one month prior to the grain quality traits evaluation. Three hundred grams of grains was sampled from each replicate. The grains were mechanically dehulled and polished using a mini-polisher. Four physical grain qualities, including percentages of brown rice (BR), head rice (HR), grain length (GL/W) and %cooking elongation increased (CE), were evaluated for the polished rice. Ten grains of paddy rice were measured using a Vernier caliper, and the GL/W ratio was calculated. The polished rice grain length (PRL) was measured using the same method. The cooking elongation of the polished rice was determined by boiling 20 grains in 5 ml of dH_2_O for ten minutes. Cooked grain lengths were measured after air-drying the grains for 1 hour. Two chemical grain qualities, amylose content (AC) and gel temperature (GT), were evaluated following the procedures described by Lanceras et al. (2000). GT is an indicator of the time required for cooking. The GT was indirectly estimated based on the alkali spreading value (ASV); higher values of ASV represent increased spreading in alkali and therefore represent lower values of GT; conversely, smaller values of ASV indicate higher values of GT.

### Genomic scanning of foreground and background

Using simple sequence repeat (SSR) markers spanned across the genome, the effects of foreground selection on linkage drag and genome background content were estimated using the graphical genotyping software GGT 2.0 (Berloo 2007) based on a specific F_2_ population type calculation. The effects of marker-assisted selection were studied on BC_1_F_2_ and pseudo-backcross progeny after recombining all target genes/QTLs (PinK + 4). Five selected BC_1_F_2_ (Additional file 2) and nine pseudo-BC_3_F_3_BILs (PinK + 4) (Additional file 3) representing nine recombined families were analyzed for genetic background recovery; 61 SSR markers showing clear polymorphism between parents were used (Additional file 1). Twenty-six markers distributed over six non-carrier chromosomes (chromosomes 2, 3, 7, 8, 10 and 12), as well as 35 SSR markers distributed over six carrier chromosomes (chromosomes 1, 4, 5, 6, 9 and 11) were used for background scanning. Furthermore, QTL-specific markers located within each QTL were developed to estimate the risk of target loss. Definitions of parameters describing % recurrent genome content (%RGC) and % donor genome content (%DGC) were calculated according to Xi et al. (2006), (Suh et al. (2009) and Suh et al. (2013) with some modification.

Donor segment link: homozygous allele similar to each donor on the same arm of the carrier chromosome.

Heterozygous segment link: heterozygous allele on the same arm of the carrier chromosome.

Donor segment unlink: homozygous allele similar to each donor on the other arm of the carrier chromosome.

Heterozygous segment unlink: heterozygous allele on the other arm of the carrier chromosome.

Donor segments on non-carrier chromosome: homozygous allele similar to each donor on non-carrier chromosomes.

Heterozygous segments on non-carrier chromosomes: heterozygous allele on non-carrier chromosomes.

Recurrent background: homozygous allele similar to the pseudo-recurrent ‘PinK3’ allele.

## Additional files

Additional file 1: **Polymorphic SSR markers used for background survey and QTL/functional markers for foreground selection using physical distance (Pseudomoleculerelease7) on carrier and non-carrier chromosomes.**

Additional file 2: **The genomic composition (average per line) of five selected BC**_**1**_ **F**_**2**_ **lines resulting from four donors: CholSub1, Xa497, RBPiQ and Bph162.**

Additional file 3: **The percentage of genome compositions (average per line) of nine selected pseudo-BC**_**3**_ **F**_**3**_ **BILs.**

Additional file 4: **Average lesion length in centimeters of pseudo-BC**_**3**_ **F**_**3**_ **BILs(PinK + 4)as well as the donor and recurrent parents when challenged with four Thai** ***Xanthomonas oryzae*** **pv. oryzae isolates.**

Additional file 5: **Pseudo-backcrossing scheme for multiple gene pyramiding based on one single backcrossing to maintain the percentage of recurrent genome content at 75% in the successive pseudo-backcrossing phase.** This novel platform facilitates the introduction of additional genes/QTLs to be pyramided into the current genotyping design. Once the desirable genotype is constructed, selfing, MAS and phenotypic selection increase the likelihood of optimizing the desirable pyramided lines.

## Abbreviations

%RGC: Recurrent genome content
%DGC: Donor genome content
BC_n_F_n_: Pseudo-backcrossing
Sub: Submergence
BB: Bacterial leaf blight
BL: Blast
BPH: Brown planthopper
MAB: Marker-assisted backcrossing
