# Supplementary material for: Pseudo-backcrossing design for rapidly pyramiding multiple traits into a preferential rice variety
Source: Rice (N Y). 2015 Feb 5;8:7. doi: 10.1186/s12284-014-0035-0 (PMC4384721; doi:10.1186/s12284-014-0035-0)
Supplement: Supplementary file 1 — Polymorphic SSR markers used for background survey and QTL/functional markers for foreground selection using physical distance (Pseudomoleculerelease7) on carrier and non-carrier chromosomes. [file 12284_2014_35_MOESM1_ESM.docx]

**Additional file 1:** Polymorphic SSR markers used for background survey and QTL/functional markers for foreground selection using physical distance (Pseudomoleculerelease7) on carrier and non-carrier chromosomes.

| **Marker on carrier chromosomes** | | | |  |
| --- | --- | --- | --- | --- |
| **Chro.** | **Physical position (Mb)** | **Marker name** | **Marker Type** | **Application** |
| 1 | 3.95 | RM84 | SSR | Background scanning |
|  | 10.84 | RM488 | SSR | Background scanning |
|  | 11.39 | RM543 | SSR | Background scanning |
|  | 24.8 | RM243 | SSR | Background scanning |
|  | 33.05 | RM212 | SSR | Background scanning |
| qBL1 | 33.06 | TBGI055578 | SNP | SNPs associate with blast resistance |
|  | 33.17 | TBGI055716 | SNP | SNPs associate with blast resistance |
|  | 33.19 | TBGI055841 | SNP | SNPs associate with blast resistance |
|  | 33.7 | RM302 | SSR | Background scanning |
|  | 34 | RM319 | SSR | Background scanning |
|  | 35.47 | RM312 | SSR | Background scanning |
| 4 | 0.69 | RM335 | SSR | Background scanning |
| *TPS* | 13.15 | RM307 | SSR | Background scanning |
|  | 16.21 | TPS | SNP | Functional marker for Bph resistance |
|  | 18.75 | RM185 | SSR | Background scanning |
|  | 27.04 | RM241 | SSR | Background scanning |
| 5  BB | 0.31 | RM122 | SSR | Background scanning |
|  | 0.44 | xa5 | SNP | Functional marker BB |
|  | 0.49 | RM159 | SSR | Background scanning |
|  | 2.01 | RM13 | SSR | Background scanning |
|  | 26.91 | RM274 | SSR | Background scanning |
| 6 | 0.28 | RM133 | SSR | Background scanning |
| qBph3 | 1.21 | 1210 | SNP | SNPs associate with Bph resistance |
|  | 1.28 | 3380 | SNP | SNPs associate with Bph resistance |
|  | 1.38 | 1380 | SNP | SNPs associate with Bph resistance |
|  | 1.38 | RM589 | SSR | Background scanning |
|  | 1.45 | RM19310 | SSR | Background scanning |
|  | 1.48 | RM586 | SSR | Background scanning |
|  | 1.61 | RM588 | SSR | Background scanning |
| Starch quality | 1.76 | RM190 | SSR | Background scanning |
|  | 1.76 | Wx | SNP | Functional marker for AC |
| Photoperiod sensitive | 6.75 | SSiia | SNP | Functional marker for GT |
|  | 9.34 | hd1 | SNP | SNPs associate with photosensitivity |
|  | 19.51 | RM541 | SSR | Background scanning |
| Sub | 28.43 | RM400 | SSR | Background scanning |
| 9 | 6.38 | Sub1C | SNP | Functional marker for submergence tolerance |
|  | 6.57 | RM464 | SSR | Background scanning |
|  | 18.56 | RM285 | SSR | Background scanning |
|  | 20.07 | RM105 | SSR | Background scanning |
|  | 22.58 | RM245 | SSR | Background scanning |
| 11 | 2.84 | RM332 | SSR | Background scanning |
|  | 5.75 | RM120 | SSR | Background scanning |
|  | 18.27 | RM209 | SSR | Background scanning |
| BB | 19.93 | RM5961 | SSR | Background scanning |
|  | 21.04 | Xa21 | SNP | Functional marker for BB resistance |
|  | 22.48 | RM206 | SSR | Background scanning |
|  | 27.67 | RM224 | SSR | Background scanning |
|  | 27.34 | TBGI453126 | SNP | SNPs associate with blast resistance |
| qBL11 | 27.53 | TBGI453598 | SNP | SNPs associate with blast resistance |
|  | 27.89 | TBGI454069 | SNP | SNPs associate with blast resistance |
|  | 28.24 | TBGI454717 | SNP | SNPs associate with blast resistance |
|  | 28.26 | TBGI454800 | SNP | SNPs associate with blast resistance |
|  | 28.8 | RM144 | SSR | Background scanning |

**Additional file 1:** (Cont.)

| **Markers on Non-carrier chromosomes** | | | | |
| --- | --- | --- | --- | --- |
| **Chro.** | **Physical position (Mb)** | **Marker name** | **Marker Type** | **Application** |
| 2 | 7.71 | RM145 | SSR | Background scanning |
|  | 22.49 | RM6843 | SSR | Background scanning |
|  | 29.59 | RM6 | SSR | Background scanning |
|  | 32.78 | RM250 | SSR | Background scanning |
| 3 | 2.45 | RM231 | SSR | Background scanning |
|  | 4.95 | RM545 | SSR | Background scanning |
|  | 9.95 | RM251 | SSR | Background scanning |
|  | 35.29 | RM514 | SSR | Background scanning |
| 7 | 5.48 | RM125 | SSR | Background scanning |
|  | 12.78 | RM214 | SSR | Background scanning |
|  | 18.13 | RM418 | SSR | Background scanning |
|  | 25.65 | RM18 | SSR | Background scanning |
| 8 | 5.11 | RM310 | SSR | Background scanning |
|  | 11.76 | RM44 | SSR | Background scanning |
|  | 19.05 | RM5982 | SSR | Background scanning |
|  | 20.38 | Os2AP | SNP | Recurrent selection on BC_1_F_1_ |
|  | 20.65 | RM223 | SSR | Background scanning |
|  | 24.72 | RM149 | SSR | Background scanning |
|  | 27.89 | RM281 | SSR | Background scanning |
| 10 | 2.62 | RM222 | SSR | Background scanning |
|  | 9.69 | RM239 | SSR | Background scanning |
|  | 13.83 | RM8201 | SSR | Background scanning |
|  | 22.44 | RM333 | SSR | Background scanning |
| 12 | 0.43 | RM415 | SSR | Background scanning |
|  | 2.43 | RM19 | SSR | Background scanning |
|  | 18.32 | RM277 | SSR | Background scanning |
|  | 19.92 | RM6022 | SSR | Background scanning |
