# Supplementary material for: Pseudo-backcrossing design for rapidly pyramiding multiple traits into a preferential rice variety
Source: Rice (N Y). 2015 Feb 5;8:7. doi: 10.1186/s12284-014-0035-0 (PMC4384721; doi:10.1186/s12284-014-0035-0)
Supplement: Supplementary file 2 — The genomic composition (average per line) of five selected BC 1 F 2 lines resulting from four donors: CholSub1, Xa497, RBPiQ and Bph162. [file 12284_2014_35_MOESM2_ESM.docx]

**Additional file 2:** The genomic composition (average per line) of five selected BC_1_F_2_ lines resulting from four donors: CholSub1, Xa497, RBPiQ and Bph162.

| **Region** | **% genome compositions of BC_1_F_2_ lines** | | | | | |
| --- | --- | --- | --- | --- | --- | --- |
|  | **Sub_92_108**^1,4/^ | **BB_17C9**^1/^ | **BB_13A6**^1^ | **BL_4D3**^1,^**^2/^** | **Bph_1H6**^1,2,3/^ | **Ave.** |
| Target gene/QTL | <0.01 | <0.01 | <0.01 | 0.29 | 0.05 | 0.07 |
| Donor segment link | 1.81 | 3.50 | 4.04 | 1.59 | 0.00 | 2.19 |
| Heterozygous segment link | 0.00 | 0.00 | 1.92 | 1.07 | 1.16 | 0.83 |
| Donor segment unlink | 0.00 | 0.00 | 2.06 | 0.00 | 2.60 | 0.93 |
| Heterozygous segment unlink | 0.00 | 1.03 | 0.00 | 2.00 | 0.00 | 0.61 |
| Target carrier chromosome (1ch) | 1.81 | 4.53 | 8.02 | 4.95 | 3.81 | 4.62 |
| Donor segments on non-target carrier chromosome (4-5 ch) | 10.70 | 19.35 | 4.66 | 0.79 | 3.05 | 7.71 |
| Heterozygous segments on non-target carrier chromosome (4-5 ch) | 0.00 | 0.52 | 6.1 | 2.49 | 0.72 | 1.96 |
| Donor segments on non-carrier chromosome (6 ch) | 9.92 | 4.69 | 3.20 | 3.64 | 0.00 | 4.29 |
| Heterozygous segments on non-carrier chromosome (6 ch) | 0.00 | 0.92 | 3.68 | 0.86 | 3.46 | 1.78 |
| Recurrent background (11 ch) | 77.57 | 67.53 | 62.62 | 80.84 | 83.64 | 74.45 |
| **% RGC** | 77.57 | 69.99 | 74.33 | 87.26 | 88.98 | 79.63 |
| **% DGC** | 22.43 | 30.01 | 25.67 | 12.74 | 11.02 | 20.37 |

^1/^=Fixed homozygous *Wx^A^* allele in four BC_1_F_1_ lines.

^2/^=Fixed homozygous aromaticallele in BL_4D3 and Bph_1H6 lines based on *aromarker* gene selected on the BC_1_F_1_ generation.

^3/^=Fixed homozygous non-photosensitive allelein Bph_1H6 on the BC_1_F_1_ generation.

^4/^=Fixed heterozygous of GT allele based on *SSiia* gene in Sub 92-108 on the BC_1_F_1_ generation.
