# Supplementary material for: Pseudo-backcrossing design for rapidly pyramiding multiple traits into a preferential rice variety
Source: Rice (N Y). 2015 Feb 5;8:7. doi: 10.1186/s12284-014-0035-0 (PMC4384721; doi:10.1186/s12284-014-0035-0)
Supplement: Supplementary file 3 — The percentage of genome compositions (average per line) of nine selected pseudo-BC 3 F 3 BILs. [file 12284_2014_35_MOESM3_ESM.docx]

| **Region** | **% genome compositions of pseudo BILs** | | | | | | | | | |
| --- | --- | --- | --- | --- | --- | --- | --- | --- | --- | --- |
|  | **1E_06** | **20A09** | **35A10** | **36C04** | **66B09** | **78A03** | **90A08** | **104A03** | **117A08** | **Ave (%)** |
| Target gene/QTL | 0.34 | 0.34 | 0.34 | 0.34 | 0.34 | 0.34 | 0.34 | 0.34 | 0.34 | **0.34** |
| Donorsegment link | 11.41 | 1.22 | 9.97 | 10.38 | 4.09 | 5.6 | 4.04 | 9.00 | 4.48 | **6.69** |
| Heterozygous segment link | 0.43 | 1.88 | 0.82 | 1.78 | 3.09 | 2.31 | 1.92 | 2.27 | 2.31 | **1.87** |
| Donor segment unlink | 3.06 | 4.19 | 0.00 | 7.41 | 4.44 | 8.31 | 5.66 | 6.16 | 4.71 | **4.88** |
| Heterozygous segment unlink | 0.98 | 1.37 | 2.01 | 2.01 | 0.00 | 0.98 | 0.00 | 0.98 | 0.98 | **1.03** |
| Donor segments on non-carrier chromosome (6ch) | 0.00 | 9.78 | 6.82 | 1.72 | 4.53 | 5.06 | 9.77 | 3.33 | 4.21 | **5.03** |
| Heterozygous segments on non-carrier chromosome (6ch) | 3.33 | 1.06 | 2.72 | 1.86 | 5.37 | 2.84 | 2.34 | 3.24 | 1.67 | **2.71** |
| Sum of donor segments (12 ch.) | 19.54 | 19.835 | 22.665 | 25.495 | 21.86 | 25.435 | 24.06 | 25.31 | 18.69 | **22.54** |
| Recurrent background (11 ch) | 75.71 | 75.85 | 71.77 | 68.85 | 69.68 | 68.43 | 71.67 | 68.19 | 76.34 | **71.83** |
| **%RGC** | **80.45** | **80.16** | **77.32** | **74.50** | **78.14** | **74.56** | **75.93** | **74.68** | **81.30** | **77.44** |
| **%DGC** | **19.55** | **19.84** | **22.68** | **25.50** | **21.86** | **25.44** | **24.07** | **25.32** | **18.70** | **22.56** |

**Additional file 3:** The percentage of genome compositions (average per line) of nine selected pseudo-BC_3_F_3_BILs.
