# Supplementary figures and images for: Pseudo-backcrossing design for rapidly pyramiding multiple traits into a preferential rice variety
Source: Rice (N Y). 2015 Feb 5;8:7. doi: 10.1186/s12284-014-0035-0 (PMC4384721; doi:10.1186/s12284-014-0035-0)

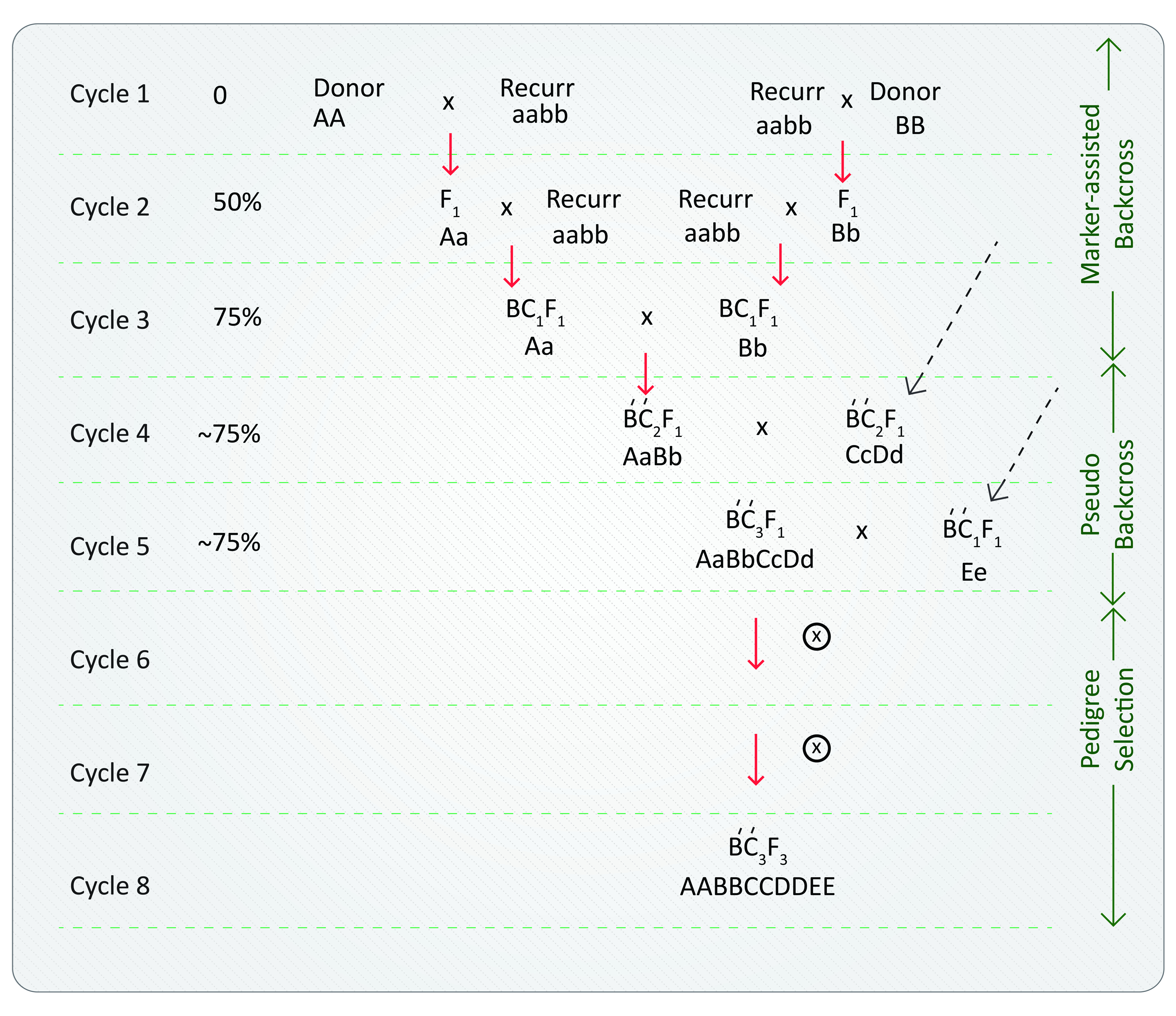

Supplement: Supplementary file 5 — Pseudo-backcrossing scheme for multiple gene pyramiding based on one single backcrossing to maintain the percentage of recurrent genome content at 75% in the successive pseudo-backcrossing phase. This novel platform facilitates the introduction of additional genes/QTLs to be pyramided into the current genotyping design. Once the desirable genotype is constructed, selfing, MAS and phenotypic selection increase the likelihood of optimizing the desirable pyramided lines. [file 12284_2014_35_MOESM5_ESM.jpeg]
